# Metachronous ureteral metastasis of a gastric adenocarcinoma: a case report and review of literature

**DOI:** 10.1186/s13256-023-04065-y

**Published:** 2023-08-11

**Authors:** Meriem Ait Alla, Tarik Chekrine, Zineb Bouchbika, Nadia Benchakroun, Hassan Jouhadi, Nezha Tawfiq, Houda Chaouki, Farida Marnissi, Mehdi Karkouri, Souha Sahraoui

**Affiliations:** 1 Department of Radiation Oncology, University Hospital Center Ibn Rochd, Faculty of Medicine and Pharmacy, Hassan II University, Casablanca, Morocco; 2 Department of Pathology, University Hospital Center Ibn Rochd, Faculty of Medicine and Pharmacy, Hassan II University, Casablanca, Morocco

**Keywords:** Ureter, Metastasis, Gastric cancer, Adenocarcinoma, Case report

## Abstract

**Background:**

Ureteral metastasis from gastric cancers are rare and can be a cause of ureteral obstruction. There have been few published case reports in the literature. In this paper, we report an additional case and a review of the literature of all the previous reported cases.

**Case presentation:**

A 67 years old North African women who was treated four years before for a gastric adenocarcinoma, presented with abdominal pain. Imaging and endoscopy showed a mural stenosis of the left ureter, without any other abnormality. Histopathology confirmed the gastric origin of the metastasis. A palliative chemotherapy was foreseen, but due to the deterioration of the general condition of the patient, she received palliative care. We have also reviewed the literature and reported the previously published cases of ureteral metastasis from gastric cancer.

**Conclusions:**

It is worth recalling that in a context of neoplasia and with the presence of signs of ureteral obstruction, it is important to keep in mind the possibility of a ureteral metastasis.

## Introduction

Ureteral metastasis from cancers are very scarce. The first reported case was in 1909 by Stow [1]. Prostate, bladder, breast, gastro intestinal cancers and lymphoma are the main primary tumors [2]. Their treatment can be challenging and the outcomes are poor.

The incidence of ureteral metastasis of gastric carcinomas is hard to estimate due to the shortage of studies. In 1931 Pressman and Elrich published a paper about metastatic tumors of the ureter according to which “true metastasis” refers to malignant cells localized in “a portion of the ureteral wall together with the absence of any neoplasm in adjacent tissues” [2]. The mechanisms of ureteral metastasis are multiple.

In this paper, we report a case of a woman treated for a gastric cancer four years before, who presented a ureteral obstruction that turned to be a ureteral metastasis of her gastric cancer. We also conducted a review of all the previous published cases of ureteral metastasis of gastric cancers.

## Case report

A 67 year-old North African women was treated in 2018 for a gastric adenocarcinoma located in the antrum. She underwent the FLOT protocol (Docetaxel, oxaliplatin, leucovorin and 5-fluorouracil) and received pre-operative chemotherapy followed by a subtotal gastrectomy with D2 lymphadenectomy. The pathological stage was ypT2N0M0. Subsequently, she received adjuvant chemotherapy. After completing the treatment, the patient underwent regular follow-up. She had no family history.

**Fig. 1:**
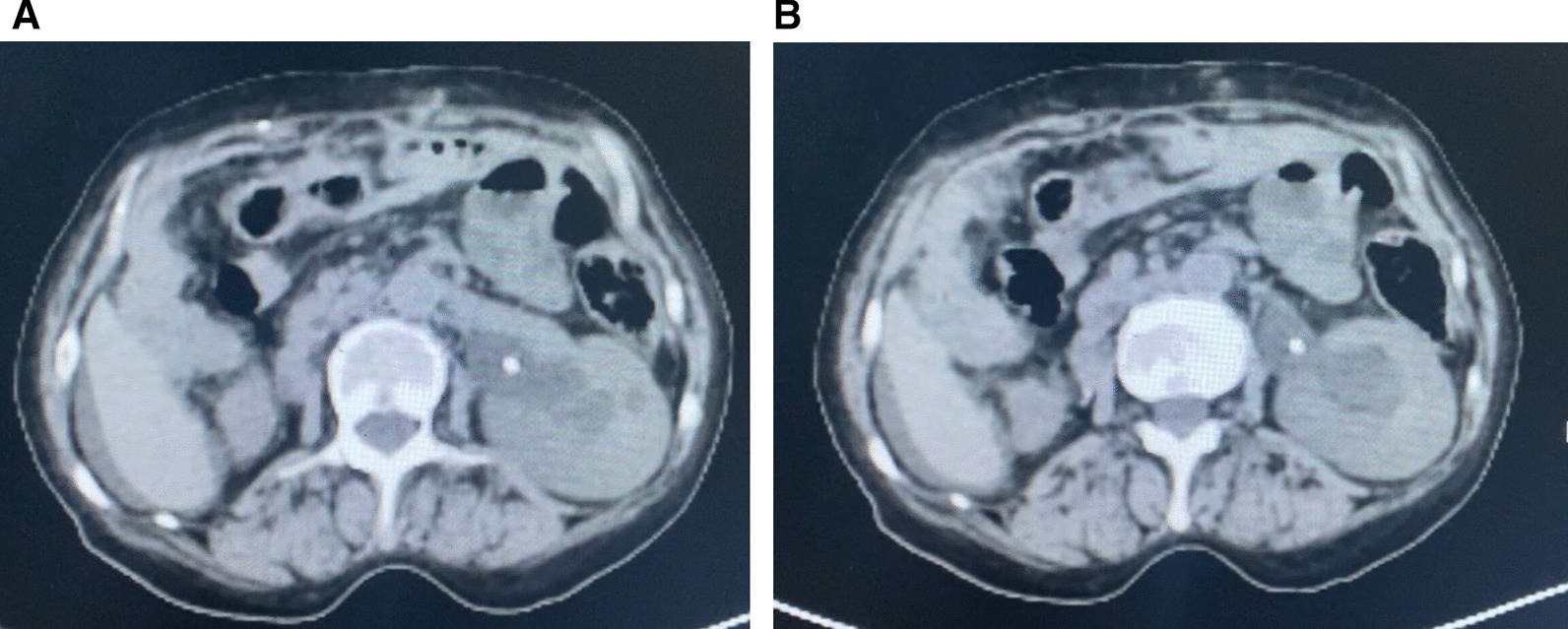
**A**, **B** Axial CT scan showing the state of the solitary left kidney with the double J stent in place

Upon follow-up, four years after, the patient complained of diffuse abdominal pain. The physical examination was normal, apart from left flank tenderness. A Computerized tomography (CT) urogram was performed, which revealed a left hydronephrosis without evidence of a ureteral stone, on a congenital solitary kidney. Ureteroscopy revealed a mural stenosis of 2 cm of the pelvic ureter. Endoscopic dilatation was performed, and a double J stent was inserted. Multiple biopsies were taken during the ureteroscopy. Another CT scan was performed Fig. 1A, B.

**Fig. 2:**
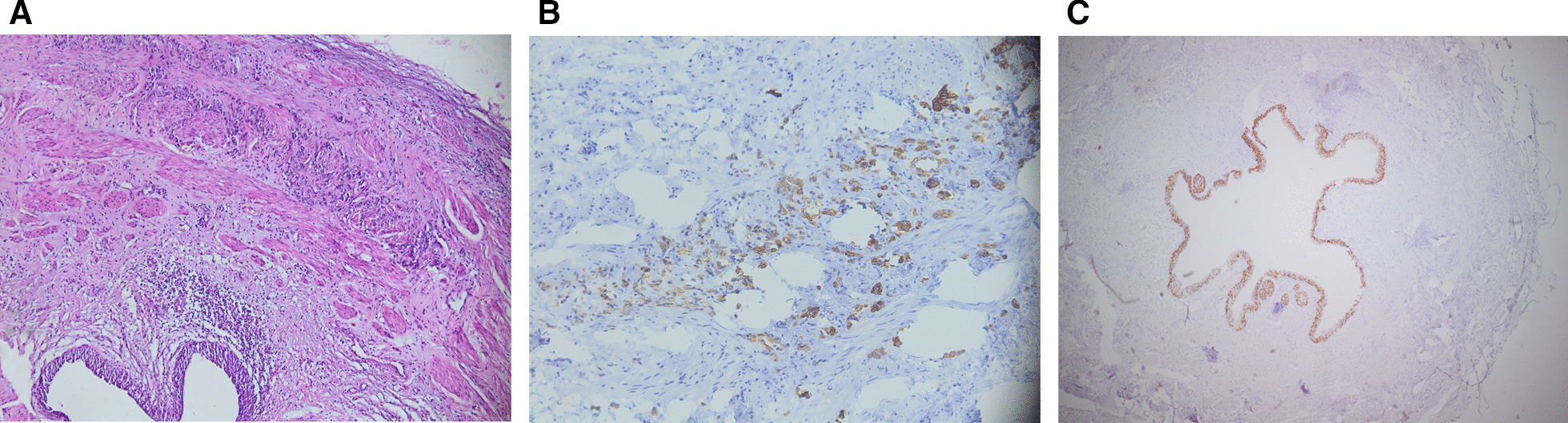
Pathological findings of the tumor. **A** infiltration of the ureteral wall by an undifferentiated proliferation (x100 magnification). **B** Positive staining for CK7 (x200 magnification). **C** Negative staining for GATA3 (x4 magnification)

Histopathology showed an infiltration of the ureteral wall by an undifferentiated proliferation Fig. 2A. Immunohistochemistry showed and expression of Cytokeratines (CK) AE1/AE3 and CK7 Fig. 2B. CK 20 and CD X2 staining were negative, as well as GATA3 Fig. 2C.

It was concluded that the proliferation was a metastasis of the previously treated gastric adenocarcinoma. HER2 (Human Epidermal Growth Factor Receptor-2) was not expressed.

A positron emission tomography scan showed no other distant metastases, particularly no abnormal activity in the abdomen or lymph node areas. However, it did reveal moderately abundant ascites.

It was decided to initiate palliative chemotherapy based on the FOLFOX protocol (bolus and continuous of 5-fluorouracil, leucovorin and oxaluplatin). However, due to the patient's deteriorating general condition, she only received one cycle of chemotherapy. Her worsening condition made it impossible to continue with the treatment. Hence, she was regularly seen by our palliative care team, to better manage her symptoms and improve her quality of life. She received analgesic treatments, injectable anti emetics to alleviate vomiting, along with nutritional support. Unfortunately, her general condition has not improved, and there was no possibility to continue the specific treatment. She died 5 months after the diagnosis was established.

## Discussion

Ureteral Metastasis of gastric adenocarcinoma is rare, but there are many published case reports. The first reported case was in 1911 by the German physician Schlangintweit [3]. Ureteral metastasis can occur from several malignancies such as breast, colon, prostate and cervix [4].

As mentioned before, ureteral metastasis has multiple mechanisms. In 1931, MacKenzie and Ratner reported three cases of metastatic growth in the ureter, in which one was of a gastric adenocarcinoma, and proposed that in metastatic growths of the ureters malignant cells can always be demonstrated in the perivascular lymphatic spaces or in the blood vessels draining the ureter [5]. Later in the same year, Presman and Ehrlich modified the definition of a true metastasis and described that true metastasis is “the demonstration of malignant cells in a portion of the ureteral wall together with the absence of any neoplasm in adjacent tissues” [2]. In our case, there was no marks of tumor surrounding the ureter, nor in the retroperitoneal space. Therefore, our case is a true metastasis according to Presman and Ehrlich definition.

The typical dissemination of malignant cells is through lymphatic and blood vessels. Other mechanisms of ureteral obstruction are more frequent. They include other mechanisms of ureteral compression by peritoneal deposits, given the frequency of peritoneal metastases in stomach cancer, or by lymph node metastasis, or in locally advanced forms, by direct extension from the primary site [6]. Another possible mechanism is malignant retroperitoneal fibrosis, which is a condition characterized by a sclerotic reaction invading the periureteral region induced by the malignant cells [7].

We conducted a research on the bibliographic data search engine Pubmed using the Mesh terms “neoplasm metastasis” AND “ureter “AND “stomach neoplasms” from 1958 until 2023. We also referred to the bibliography of the articles found. Table 1 summarizes the 54 published cases of ureteral metastasis from gastric cancers (including our case) until this day.

**Table 1:** Summary of published case of ureteral metastasis of gastric cancer

Author	Year	Number of cases	Age	Gender	Symptoms	Localisation	Chronology	Treatment	Survival
Mackenzie and Ratner [5]	1931	1	61	M	Abdominal pain	Body of stomach	Synchronous	–	–
Bartels et al. [8]	1933	1	–	–	–	–	–	–	–
Fergusson et al. [9]	1944	1	39	M	Abdominal pain	Pyloric antrum	Synchronous	Surgical exploration	6 weeks
Presman et al. [2]	1948	1	41	M	Abdominal pain	Pylorus	Synchronous	Nephrectomy	One year
Stearns et al. [10]	1958	1	-	M	Hematuria	–	Metachronous (one year and 5 months)	Uretero Nephrectomy	4.5 months
Fitch et al. [11]	1976	1	72	M	Abdominal pain	Pylorus	Synchronous	Nephrectomy and partial ureterectomy	11 months
Babain et al. [12]	1980	12	–	–	–	–	–	–	–
Jibiki et al. (in Japenese) [13]	1995	23	Mean age: 52	Sex Ratio: 14 M/9F					
Ushida et al. [14]	1999	3	25 38 50	F F F	Upper abdominal discomfort	–	Synchronous	Chemotherapy:methetrexate + 5-fluorouracil	–
Heesakkers et al. [15]	1999	1	69	M	Pain in the left lower abdomen	–	Metachronous (9 months)	Double J stent, palliative care	
Vilaseca Cabo et al. [16]	1999	2	82 86	M M	Fatigue and anorexia –	Antrum	Metachronous (6 months) Metachronous (6 years)	Nephroureterectomy Nephroureterectomy	– –
Shimoyama et al. [17]	2000	1	51	F	Right flank pain	Upper part of the stomach	Synchronous	Chemotherapy Cispatine + 5 Fluorouracil	One year
Yeh et al. [18]	2008	1	66	M	Pain in lower abdomen	Antrum	Metachronous (3 years)	Nephro-uterechectomy and bladder cuff excision	3.5 months
Bisof et al. [19]	2009	1	50	M	Asymptomatic	Antrum	Metachronous (4 years)	Systemic chemotherapy	Alive (a year after treatment)
Tsung and al [20]	2020	1	55	M	Right flank pain	–	Synchronous	Bilateral percutaneous nephrostomy + chemotherapy	–
Guitynavard et al. [21]	2022	1	80	M	Bilateral flank pain and fever	–	Metachronous (1 year)	Surgery bilateral distal ureteral resection and re-implant	–
Kawaguchi et al. [22]	2023	1	63	M	Asymptomatic	–	Metachronous (7 years)	Radiotherapy 50.4 Gy (1.8 Gy/Fraction) Chemotherapy: RAM protocol + PTX Nivolumab	4 years and 3 months
Our case	2023	1		F	Abdominal pain	Antrum	Metachronous (4 years)	Insertion of a double J stent; palliative chemotherapy (1 cycle); palliative care	5 months

Jibiki et al. [13] made a review in Japanese in 1995 of the previous cases published in Japan, which is the longest series of cases, concerning 23 cases. The mean age of the patients was 52 years. More than a third of patients (41%) had been previously operated for gastric cancer. There was no sex predominance and 44% of the cases occurred bilaterally. Poorly differentiated adenocarcinoma and signet-ring carcinoma were the most common histological type.

According to our literature review, among the cases where sex was indicated, 34% were female, whereas 66% were male. Age ranged between 25 and 86, with a mean age above 50 years. There was no predominance regarding the chronology of the occurrence of metastasis, as the percentage was 50% for both metachronous and synchronous occurrence

As for symptoms of ureteral metastasis from gastric cancers, mostly flank pain, they can be the first symptoms of an asymptomatic gastric cancer [17, 19]. Other symptoms include abdominal pain, hematuria or oligouria.

No effective therapy for this condition has been judged effective. Treatment mostly depend on the general condition of the patient, the presence of other metastasis and the extent of the ureteral obstruction and feasibility of a surgery. In four of the seven cases reported before 1980, for which the treatment modality was mentioned, all patients underwent surgery: surgical exploration or nephrectomy. While in case reports published in the last 30 years, patients received systemic treatments. There was only one case treated with Radiotherapy with a dose of 50.4 Gy [22].

Given that this condition often designates a very advanced stage of the gastric cancer, and that the best overall survival of patients with metastatic gastric cancer is 13.8 months in patients with HER2 expression treated with Trastuzumab plus chemotherapy [23], the prognosis is usually poor and the survival doesn’t exceed one year. The longest survival span is 4 years and 3 months [22]

## Conclusion

It is important to keep in mind, in a context of neoplasia and with the presence of signs of ureteral obstruction, the possibility of a ureteral metastasis. As these can occur many years after treatment of the primary cancer.

## Data Availability

Not applicable.
